# Performance comparison of LUR and OK in PM_2.5_ concentration mapping: a multidimensional perspective

**DOI:** 10.1038/srep08698

**Published:** 2015-03-03

**Authors:** Bin Zou, Yanqing Luo, Neng Wan, Zhong Zheng, Troy Sternberg, Yilan Liao

**Affiliations:** 1 School of Geosciences and Info-Physics, Central South University, Changsha. 410083, China; 2 Shanghai Key Laboratory of Atmospheric Particle Pollution and Prevention (LAP^3^), Shanghai, 200433. China; 3 Department of Geography, University of Utah, Salt Lake City. UT 84112, USA; 4 School of Geography and Environment, Oxford University, Oxford, UK; 5 State Key Laboratory of Resources and Environmental Information System, Chinese Academy of Sciences, Beijing. 100001, China

## Abstract

Methods of Land Use Regression (LUR) modeling and Ordinary Kriging (OK) interpolation have been widely used to offset the shortcomings of PM_2.5_ data observed at sparse monitoring sites. However, traditional point-based performance evaluation strategy for these methods remains stagnant, which could cause unreasonable mapping results. To address this challenge, this study employs ‘information entropy’, an area-based statistic, along with traditional point-based statistics (e.g. error rate, *RMSE*) to evaluate the performance of LUR model and OK interpolation in mapping PM_2.5_ concentrations in Houston from a multidimensional perspective. The point-based validation reveals significant differences between LUR and OK at different test sites despite the similar end-result accuracy (e.g. error rate 6.13% vs. 7.01%). Meanwhile, the area-based validation demonstrates that the PM_2.5_ concentrations simulated by the LUR model exhibits more detailed variations than those interpolated by the OK method (i.e. information entropy, 7.79 vs. 3.63). Results suggest that LUR modeling could better refine the spatial distribution scenario of PM_2.5_ concentrations compared to OK interpolation. The significance of this study primarily lies in promoting the integration of point- and area-based statistics for model performance evaluation in air pollution mapping.

Numerous studies have identified the negative impact of fine particulates (PM_2.5_) on respiratory health and human mortality^1^,^2^. However, understanding and monitoring harmful particulates PM_2.5_ has encountered several challenges so far, among which the most serious one is the insufficient PM_2.5_ data due to the expensive equipment and sparsely distributed field monitoring sites. This leads to difficulties in detecting the spatial characteristics and spatial-temporal dynamics of PM_2.5_ pollution and designing effective control strategy.

Several methods have been developed over the last decade to strengthen PM_2.5_ field monitoring which is critical in understanding global PM_2.5_ exposure. Efforts mainly include remote sensing image retrieval, air dispersion modeling, spatial interpolation, and land use regression (LUR) modeling^3^. However, investigation must explore advantages and shortcomings to determine the most effective approach for a specific situation.

While remote sensing techniques are able to retrieve particulate distribution over an image area-based on the relationship between aerosol optical depth (AOD) and PM_2.5_ concentration, the effectiveness is reduced when image acquisition phase-in fails. The limited spatial resolution (i.e. hundreds to thousands of meters) also makes it difficult to derive detailed PM_2.5_ spatial distribution characteristics in urban environments^4^. Similarly, air dispersion models can be used to simulate the PM_2.5_ concentration at preset receptors (i.e. grid points in this study) with various resolutions and coverage by using boundary layer turbulent diffusion theories and aerochemical theories. However, it requires copious data (e.g. emission, meteorological and terrain data) for hypothesis of the diffusion mode which makes it difficult to implement^3^,^5^,^6^,^7^.

Relatively, LUR modeling and Ordinary Kriging (OK) interpolation are two popular methods for mapping PM_2.5_ concentration based on the sparsely distributed observation data in diverse applications^8^,^9^,^10^,^11^. LUR modeling can produce PM_2.5_ concentration surfaces at fine resolutions by linking geographic elements with PM_2.5_ observation data using the least square method^12^. OK interpolation is suitable for PM_2.5_ concentration mapping based on the observation data with normal distribution and is the preferred unbiased geo-statistical technique in air pollution interpolation^13^. Unfortunately, implications of both LUR and OK methods are also limited by their inherent defects^14^. Shortcomings in unclear driving factors, non-standard predictor variable selection and poor time-space migration generally limit LUR model's effectiveness^9^. OK interpolation usually fails to produce PM_2.5_ surface in the regions with sparse or missing data and is prone to over-amplify extreme variations due to its reliance on a single factor^8^. Consequently, accurate performance evaluation of LUR modeling and OK interpolation is particularly important for reliable air pollution mapping.

While studies have attempted to promote this work through comparing the performance of LUR, Kriging and air dispersion modeling in estimating PM_10_ concentration^15^, further improvements are still needed. Because model performance in these comparative studies was largely determined by similarities of causal mechanisms on air pollution concentrations between locations of test sample sites and training sample sites^15^,^16^,^17^. Model reliability is therefore dependent on test sites selected that are subject to evaluation errors^18^.

Information entropy, an area-based statistic indicator that was originally designed to describe the even spatial distribution of energy, has been increasingly used to evaluate the richness of image information. Since air quality concentration varies over space, information entropy has the potential of reflecting this variation based on the raster map of air quality concentrations^19^,^20^. Compared to traditional point-based statistics, information entropy is an effective index that can uniquely and objectively measure the information amount of a map and evaluate the capacity of this map in disclosing variation details of an element^21^,^22^.

This study therefore employed area-based information entropy along with traditional point-based statistics to evaluate the performance of LUR modeling and OK interpolation in mapping PM_2.5_ concentration in Houston from a multidimensional perspective. In order to better understand the meaning of information entropy values, an external profile analysis is also implemented.

As a large industrialized region in southeast Texas, the Houston metropolitan area covers 10 counties and 26,060 km^2^ (Figure 1). In this study the city serves as a representative urban environment with documented high PM_2.5_ pollution rates. Prior works estimated a mean annual particulate concentrations in Houston that range from 9.87 μg/m^3^ (minimum) to 14.24 μg/m^3^ (maximum) in the metropolitan area.

In the flat landscape, industrial and traffic emissions are the main pollutant sources in the multi-county area of 6 million residents in Houston metropolitan area according to U.S Environmental Protection Agency (EPA)^23^. Therefore, factors that contribute to Houston's PM_2.5_ pollution could be land-use type, road traffic, population distribution and geographic elements that represent location and climatic characteristics.

As a result, data used for LUR modeling in this study include the annual PM_2.5_ concentration at 17 monitoring sites (10 of them locate in Harris County) from the U.S. EPA's Air Quality System Technology Transfer network^24^. These PM_2.5_ concentrations are nearly distributed as normal fashion. Air quality monitoring on these sites complies with EPA's federal reference standard or federal equivalency standard, thus providing valid data for taking official air pollution measurements and quality assurance plans^25^. Land cover map with a spatial resolution of 30 m, road networks and demographic census data are respectively from the U.S. National Land Cover Database^26^, the Environmental Systems Research Institute (ESRI) nationwide street and geocoding databases^27^, and the U.S. Census database^28^.

## Results

### PM_2.5_ concentration map

Figure 2 shows the spatial distribution of annual PM_2.5_ concentrations in Houston metropolitan area produced by methods of LUR model and OK interpolation. Significant differences in PM_2.5_ concentrations can be observed across the covered counties from Figure 2 and confirmed by Figure 3. For the LUR model based map, high concentrations of simulated PM_2.5_ (>10 μg/m^3^) were found in urban Harris County whereas the surrounding suburban counties have lower concentrations (<10 μg/m^3^). In conjunction with Figure 3, this presents an obviously gradient of PM_2.5_ concentration in Houston from Harris to the surrounding areas. For the OK interpolation result map, the interpolated PM_2.5_ concentrations reflected a clear zoning distribution with high levels of pollution (>10 μg/m^3^) in central and east Harris county followed by northeast and southwest Houston. Furthermore, comparison of Figure 2a/3a and Figure 2b/3b obviously demonstrates that the LUR simulated PM_2.5_ concentrations showed a higher level of details and smoother variations than OK interpolated results.

### Performance comparison based on point-based statistics

The performance of the LUR model and OK interpolation in PM_2.5_ concentration estimation is evaluated by using the point-based statistics including absolute error, error rate, *RMSE*, and paired *T*-test. These statistics are calculated by using the typical N-1 cross validation strategy. Results listed in Table 1 show that the error rates of both LUR simulated- and OK interpolated PM_2.5_ concentrations varied among monitoring sites. Whilst the absolute minimum and maximum errors of LUR simulated PM_2.5_ concentrations were 0.02 ug/m^3^ at site 9 and 2.04 μg/m^3^ at site 15/16 with an average absolute error of 0.70 μg/m^3^, that of the OK interpolated PM_2.5_ concentrations were 0.01 μg/m^3^ at site 15 and 2.07 μg/m^3^ at site 13 with an average absolute error of 0.80 μg/m^3^. Moreover, LUR model had an overall higher accuracy of simulated PM_2.5_ concentrations compared to the OK interpolated ones although the paired *T*-test confirmed insignificant difference in site-based error rates between these two methods at *P* = 0.65. The LUR simulated- and OK interpolated PM_2.5_ concentrations had respectively 5 and 7 sites with absolute error >1.00 μg/m^3^. The maximum error rates of these two methods were 15.95% and 20.34% with an average error rate of 6.13% and 7.01%, respectively. In addition, the *RMSE* evaluation results of LUR simulated and OK interpolated PM_2.5_ concentrations (i.e. 0.89 and 1.00, respectively) were also consistent with those based on the absolute error and error rate.

### Performance evaluation based on area-based information entropy

Table 2 displays the values of information entropy and the related statistics calculated from the spatial distribution maps simulated by LUR model and interpolated by OK. The information entropy values (i.e. LUR: 7.79 vs. OK: 3.63) in Table 2 indicate that LUR model outperformed OK interpolation in illustrating detailed spatial variations of PM_2.5_ concentrations across the Houston metropolitan area. The reliability of information entropy was echoed by the maximum, minimum and average PM_2.5_ concentrations simultaneously shown in Table 2. Specifically, the LUR model generated PM_2.5_ concentrations (9.57–13.52 μg/m^3^) were closer to the actual ground observation values (9.87–14.24 μg/m^3^) than did by OK interpolation (which ranges from 10.08–12.93 μg/m^3^). Additionally, profile analysis results in Figure 4 further confirmed above findings of information entropy evaluation. It can be observed that, along all four directions, the PM_2.5_ concentrations interpolated by the OK method almost first demonstrated an increasing trend and then gradually decreased, while those simulated by LUR model at the same local sites were relatively lower and stable at two ends but higher and fluctuated in the middle. This difference suggests that the spatial distribution scenario of PM_2.5_ concentrations could be better refined by LUR modeling rather than by OK interpolation.

## Discussion

This study explored the differences in spatial distributions of PM_2.5_ concentrations between LUR model and OK interpolation by comprehensively using point-based statistics and area-based information entropy for the first time. We found that, based on point-based statistics, the two methods produce similar results. However, highlighted significant differences were observed between the two methods based on area level information entropy and confirmed the better performance of LUR relative to OK. Our findings provide new insights for future air pollution research.

The optimal adjusted LUR model in this study has a fitting *R^2^* of 0.69, which is much higher than that of the OK method (*R*^2^ = 0.38) as well as the results of previous studies (e.g. London, 0.45 to 0.60^29^; 0.56, 0.73 and 0.50, northern Europe^30^; Germany, 0.17^31^). This study applied backward Multiple Linear Regression (MLR) method^30^,^32^,^33^ to achieve the best LUR model fitting. Due to the limited number of PM_2.5_ monitoring sites in the Houston metropolitan area, this study utilized empirical LUR variable values and sampling-site numbers to screen individual modeling variables^34^,^35^,^36^ and the strategy widely used in previous studies^37^,^38^. Variables of land use type and road traffic with strong prediction capacity are screened first. Population distribution and variables about distance to sea are then incorporated for model adjustments. Because Houston's PM_2.5_ pollution is primarily from diesel emission, oil vehicles, road dust, barbeque, and wood burning^23^, Harris County which is highly urbanized and industrialized experiences relatively higher PM_2.5_ concentration, while surrounding areas which are characterized by agricultural land use and fewer road networks have relatively lower PM_2.5_ concentrations. This is reflected by the LUR model simulated result, which shows a decreasing trend from Harris County to surrounding areas. It also confirms that the simulation result of the LUR model is closer to the real PM_2.5_ spatial distribution compared to that of the OK interpolation as shown by the statistics in Table 1, while the PM_2.5_ annual concentration of OK interpolation was zonally distributed in Houston. And, high concentration areas include the central eastern region of Harris County and the northeast and southwest regions of Houston.

The point-based statistics validation demonstrated no significant differences between the results from LUR model and OK interpolation. However, the LUR model achieved slightly better simulation accuracy than the OK interpolation (e.g. RMSE: 0.89 vs. 1.00). Given the fact that the quality of OK interpolation is dependent on the distribution of monitoring sites, the validation precision of OK interpolated PM_2.5_ concentrations at different monitoring sites would certainly vary, with poorest results being at the boundary area due to the insufficient observation data. Inversely, considering more factors such as land use, traffic, population, we believe LUR model is more reliable than OK interpolation, especially for the area without abundant observed PM_2.5_ concentrations but sufficient relevant auxiliary factors. Additionally, the point-based statistics validation process of LUR model and OK interpolation in this study is based on the typical N-1 cross validation strategy and should be the ‘best’ one we can use with discrete monitoring sites.

Furthermore, area-based information entropy evaluation revealed significantly different results between the LUR model and OK interpolation. The annual PM_2.5_ concentrations simulated by the LUR model have more spatial variations (greater information entropy values) than the OK interpolation. This is because LUR model integrates additional influencing factors that are closely related with the emission and diffusion of PM_2.5_, such as land use, road traffic, and climatic indicators. These factors strengthened the ability of LUR model in revealing the concentration variations through distinguishing the surrounding geographic differences of divergent positions, especially for areas with limited monitoring sites. These two advantages increased the information richness of the LUR simulated map and reflected the real-world scenario of PM_2.5_ concentration. These factors re-confirmed the superiority of information entropy in evaluating an air quality map's capacity in disclosing variations, which could not be achieved by previous air quality mapping studies based on traditional point-based statistics^7^,^18^,^39^. According to the urban development pattern (e.g. Harris County is with high volume of traffic and is also the industrial and economic center) and PM_2.5_ sources in Houston, we believe the evaluation result based on LUR model is more reliable than OK interpolation.

Like previously reported studies with data from few monitoring sites (i.e. minimum site number is 13)^40^,^41^, while satisfactory results have been achieved with the data collected from 17 monitoring sites in this study, issues on monitoring sites and the predictor selection still need to be addressed in the future. PM_2.5_ concentration estimation with higher accuracy could be achieved with more monitoring sites. Moreover, while this study established a significant LUR model at an acceptable accuracy level using MLR without overestimates, the model's performance definitely could be further enhanced by involving more predictors under sufficient monitoring sites that are evenly distributed in space.

In summary, findings in this study imply that although the point-based statistics evaluation could accurately reflect a model's performance in mapping air pollution concentration, its evaluation result is often limited by test site locations and their spatial distribution. In regions with densely centralized test sites and training sites, point-based statistics evaluation methods may overestimate the model accuracy (i.e. better or worse accuracy), and vice versa. Therefore, except for point-based statistics evaluation, the area-based information entropy evaluation proposed in this study is important and necessary for more comprehensive and accurate assessment of the air pollution concentration maps. In other words, the information entropy evaluation clearly confirms that LUR model is more accurate in representing the spatial distribution of annual PM_2.5_ concentrations of Houston metropolitan area than the OK interpolation in this study. Additionally, this study implies that the utilization of information entropy is a new measure to effectively evaluate the performance of other exposure models such as dispersion modeling, LUR modeling, and remote sensing based models, for which the spatial resolution is better than OK interpolation. And this could greatly enhance the reliability of findings for future environmental health studies.

## Methods

The methodology of this study is composed of three parts: LUR modeling, OK interpolation, and performance comparison between LUR and OK (Figure 5).

### LUR modeling

LUR modeling links the air pollution concentration at a monitoring site with other geographic characteristics of that monitoring site. The modeling is composed of variable extraction and screening, regression model building, and model validation. The variable extraction and screening include selecting geographic elements and extracting characteristic variables of geographic elements.

Considering experiences from previous LUR studies^10^,^11^,^30^,^32^,^33^,^42^,^43^,^44^ and PM_2.5_ pollution sources in Houston, this study utilizes annual PM_2.5_ concentrations as the outcome variable and develops predictors of various geographic elements including land use type (*X*_1_), road length (*X*_2_), distance to road (*X*_26_), population density (*X*_31_), house density (*X*_32_), and distance to sea (*X*_41_). Among them, the “measured values” of predictors with spatial scaling effect are extracted at 100 m, 300 m, 500 m, 800 m, 1000 m, 1500 m, 2000 m, 2500 m, 3000 m, 3500 m, 4000 m, 4500 m and 5000 m buffering radius due to the unclear ‘spatial scale dependency’^33^,^42^,^45^. Land use types are reclassified as forest (*X*_11_), open space (*X*_12_), medium-density urban (*X*_13_), high-density urban (*X*_14_) and barren land (*X*_15_) with the 11 initial land use types provided by United States Geological Survey. Road traffic data in this study includes highway (*X*_21_)、major road (*X*_22_)、local road (*X*_23_)、minor road (*X*_24_) and other road (*X*_25_). The entire process is implemented with ArcGIS 10.0.

To screen out effective predictors appropriate for LUR modeling in Houston, Pearson coefficient values between all predictors and annual PM_2.5_ concentration are calculated with SPSS 19.0. For predictors with spatial scaling effect, the optimal spatial scale of each predictor is defined as the one with calculated maximum Pearson coefficient in a scale range of 100 m to 5000 m. Consequently, the final predictors screened out for LUR modeling in this study are area fraction of land use type including *X*_11_-5000, *X*_12_-100, *X*_13_-100, *X*_14_-800, *X*_15_-3000; road traffic including *X*_22_-100 m, *X*_23_-300 m, *X*_24_-3000 m, *X*_25_-1500 m and *X*_26_; and others including *X*_31_-3000 m, *X*_32_-1000 m, *X*_41_.

A predictor-based regression model is established by using a multiple linear regression (MLR) equation (i.e. Equation 1) in this study. The equation is shown as
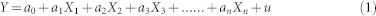
where *Y* is the annual PM_2.5_ concentration, Xdenotes independent predictors, *a_0_* is a constant, *a_1_* to *a_n_* are the regression coefficients for each predictor *X, respectively*, and *u* is the random error. An equation group is composed of *n* groups of observed values *Y_i_, X_1i_, X_2_, X_3i_, …, i* = 1,2,3..., *n*.

Area fraction of land use type and road traffic are the two major influencing factors of annual PM_2.5_ concentration in the Houston area. Thus, this study starts with backward MLR by using the respective type of predictors under an optimal spatial scale as inputs to establish the preliminary optimal models (i.e. model with highest fitting *R^2^*) with SPSS 19.0. Thereafter, another round of backward MLRs is conducted for these preliminary optimal models by adding predictors such as population density, house density and distance to sea. As a result, the finalized LUR model is built as *Y*_Conc_ = *X*_13-100_ + *X*_31-3000_ + 8.357 with significant coefficients at *p* < 0.05. The adjusted *R*^2^ of this finalized model is 0.69 with VIF values less than 10 to ensure non-multicollinearity.

Using the finalized LUR model, a continuous surface of annual PM_2.5_ concentrations at the resolution of 3 km × 3 km (Figure 2) within the study area is generated with ArcGIS 10.0 taking into account the point-based high computational cost and spatial similarity of predictors within certain spatial scales (i.e. buffering area size). Specifically, grid points with the 3 km interval across the entire study area are pre-set firstly; then the ‘measured values’ of predictors in the finalized LUR model at these pre-set points are extracted and used to calculate the annual PM_2.5_ concentrations at each pre-set point; these high density estimated PM_2.5_ concentrations are used to produce the distribution map of annual PM_2.5_ concentrations in the end.

### OK interpolation

OK interpolation refers to the linear unbiased optimal estimation of unknown points according to the structural features of known sample points^46^. When the regional variable *Z*(*x*) is a constant (m) with unknown mathematical expectations, the OK method can be used for spatial interpolation. The interpolation formula is stated as
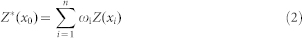
where *Z*(*x_0_*) is the value of an unknown sample point, *Z*(*x_i_*) is the value of a known sample point surrounding the unknown sample point, and Z*(*x_i_*) is the unbiased estimation of *Z*(*x_i_*) (i.e. E[Z*(x_0_) − Z(x_0_)] = *0*). *ω_i_* is the weight of the *i^th^* known sample point to the unknown sample point and 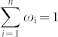 where *n* is the amount of known sample points.

For the process of OK interpolation, an exploratory data analysis is firstly conducted on the training sample data of 17 monitoring sites within the Houston metropolitan area and external 4 expanding sites outside Houston metropolitan area to determine whether the data follow a normal distribution or are spatially correlated or not. Then, a continuous prediction map of annual PM_2.5_ concentration is produced using the ‘Spatial Interpolation’ wizard of ArcGIS10.0. We did not use trend removal because of the relatively smooth variation of PM_2.5_ concentration across these monitoring sites (i.e. 17 + 4). Considering the sparse distribution of monitoring sites in the study area, the searching number of neighborhood points is set as 4.

### Point-based statistics calculation

Point-based statistics including absolute error, relative error and root-mean-square error (*RMSE*) are employed to validate methods of LUR model and OK interpolation in this study by using the commonly N-1 cross validation strategy, which is suitable for limited data samples^12^,^15^. Following this, this study divides the 17 monitoring sites across the study area into 16 training sites and 1 validation site. The absolute error represents the deviation direction and size of the simulated/estimated concentration from the observed concentration. Relative error and *RMSE* represent the deviation degree of the simulated/estimated concentration from the observed concentration, which reflect the reliability of the estimation result of the model. The three error indices are calculated according to equations (3)–(5) with larger values indicating lower model accuracy.
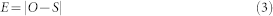

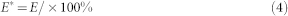

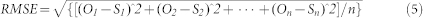
where *E*, E*, and *RMSE* respectively represent the absolute difference, relative error (i.e. error rate) and the root-mean-square error between observed concentration and estimated concentration. *O* is observed concentration, *S* is simulated/estimated concentration and n is sample size.

### Area-based information entropy

“Entropy” is an indicator that was originally designed to describe the even spatial distribution of energy^19^. It has been expanded to indicate the richness of information in information theories. Given *v* = {*X_1_*, *X_2_*,..., *X_n_*}, suppose the probability of 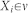 is *ρ_i_* = *P*(*X_i_*), the information entropy of *v* can be defined as:
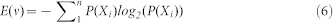
where *X_i_* represents the pixel of an image and *P(X_i_)* is the probability of occurrence of *X_i_*. The more heterogeneous *X_i_* is, the larger the information entropy of the image will be, indicating more details of the spatial pattern.

In this study, information entropy is developed to depict the ability of LUR model and OK interpolation methods in mapping the variation of the annual PM_2.5_ concentrations over the entire study area. Specifically, the distribution maps of annual PM_2.5_ concentration are firstly produced and reclassified with natural break points. Then, the number of raster grids at each class are summed and divided by the total grid number of the raster map to calculate the probabilities *P(X_i_)*. These probabilities are finally used to compute the value of information entropy according to equation (6). The calculations of information entropy for raster maps from LUR model and OK interpolation are similar and both are implemented with the modules of spatial interpolation and algebraic computation in ArcGIS 10.0.

**Figure 1:**
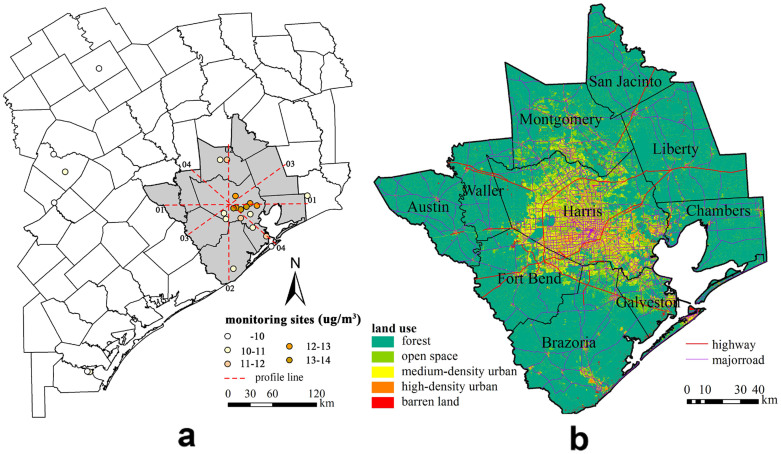
A schematic map of study area created with the basic mapping function of ArcGIS (version 10.0): (a) monitoring sites distribution, (b) distribution of major geographical elements across Houston metropolitan.

**Figure 2:**
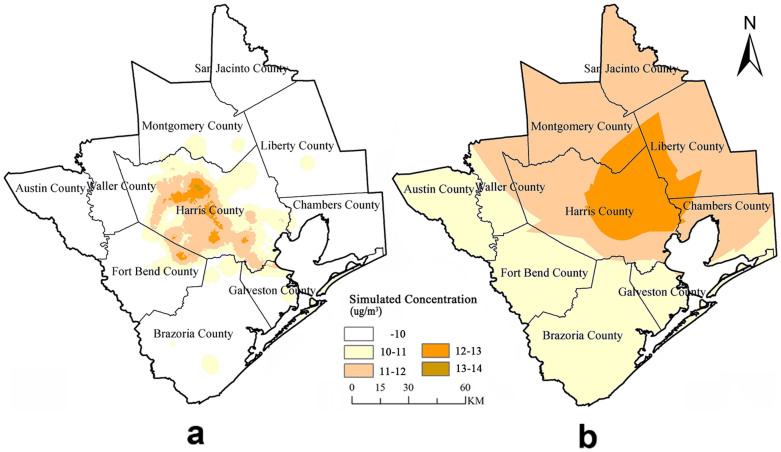
Spatial distribution map of PM_2.5_ concentration in Houston metropolitan area produced by LUR model (a) and OK interpolation (b) with the spatial analysis and geostatistical function of ArcGIS (version 10.0).

**Figure 3:**
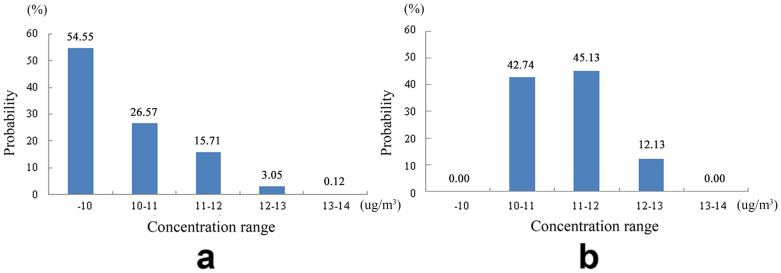
Statistic histograms of PM_2.5_ concentrations illustrated in spatial distribution map from LUR model (a) and OK interpolation (b).

**Figure 4:**
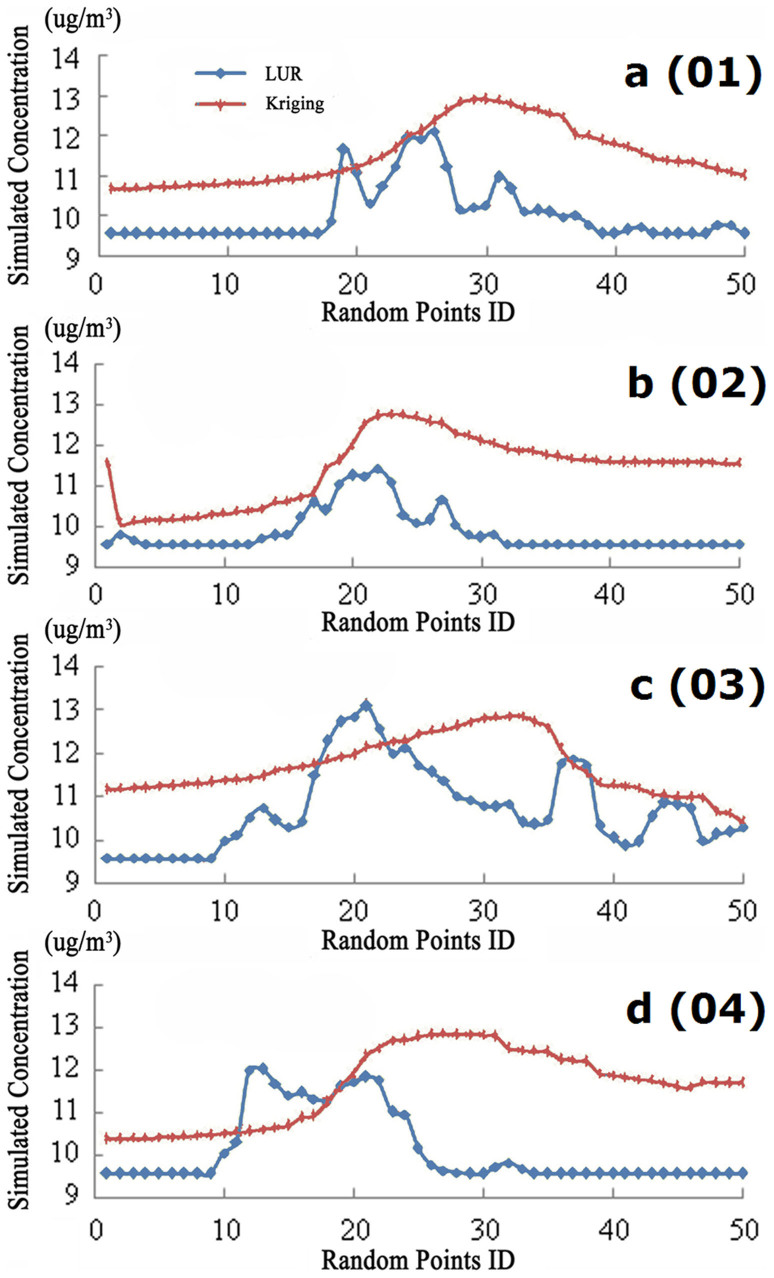
Variations of PM_2.5_ concentrations produced by LUR model and OK interpolation at four direction profiles in Houston metropolitan area: east-west a (01), south-north b (02), southeast-northwest c (03) and southwest-northeast d (04).

**Figure 5:**
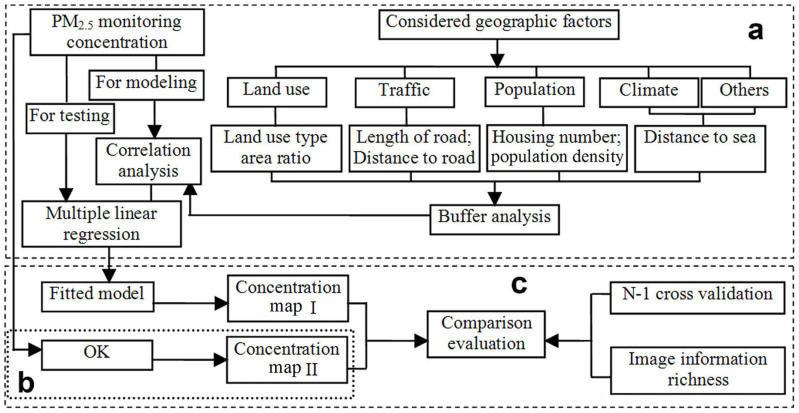
Framework of study procedure including LUR modeling (a), OK interpolation (b), and performance comparison between LUR and OK (c).

Additionally, a four-direction criterion (Figure 1), namely, east-west (01), south-north (02), southeast-northwest (03) and southwest-northeast (04) are employed to further confirm the necessity of area-based information evaluation considering factors that possibly caused the heterogeneity of PM_2.5_ concentrations. In each direction, PM_2.5_ concentrations at 50 randomly distributed sites are separately simulated and interpolated by LUR and OK methods.

## Author Contributions

B.Z. designed and performed the majority of experiments and data analysis, as well as coordinated and wrote the manuscript. Y.Q.L. participated in experimental designs and data analysis, and drafted the manuscript text. N.W., T.S., Z.Z. and Y.L.L. contributed to writing the manuscript. All authors reviewed and approved the manuscript.

## Figures and Tables

**Table 1:** Point-based statistics of observed and simulated annual PM_2.5_ concentrations. Paired *T* test is designed to test the significance of difference in error rates between LUR and OK in this table

Site ID	Obser. (O) (μg/m^3^)	LUR				OK		
		Simu. (S) (μg/m^3^)	Error (E) (μg/m^3^)	Error rate (E*) (%)		Simu. (S) (μg/m^3^)	Error (E) (μg/m^3^)	Error rate (E*) (%)
1	10.18	11.71	1.52	14.97		9.93	0.25	2.46
2	9.87	9.57	0.30	3.04		11.10	1.22	12.41
3	11.44	11.30	0.14	1.23		10.35	1.09	9.52
4	10.58	11.58	1.00	9.47		10.98	0.40	3.74
5	12.44	11.12	1.32	10.60		12.59	0.15	1.19
6	10.36	9.76	0.61	5.85		11.11	0.74	7.17
7	10.41	10.96	0.55	5.27		11.57	1.16	11.17
8	12.70	11.98	0.72	5.66		12.19	0.51	4.03
9	11.11	11.13	0.02	0.22		11.54	0.43	3.86
10	13.67	13.42	0.25	1.86		12.64	1.03	7.56
11	14.24	14.00	0.24	1.68		12.23	2.02	14.15
12	13.04	12.68	0.37	2.81		12.26	0.78	6.01
13	10.16	11.12	0.95	9.38		12.23	2.07	20.34
14	11.85	11.74	0.11	0.92		11.22	0.62	5.24
15	12.78	10.74	2.04	15.95		12.77	0.01	0.11
16	12.42	12.00	0.41	3.34		12.43	0.01	0.12
17	10.88	9.57	1.31	12.05		11.98	1.09	10.04
**Average**	**11.66**	**11.43**	**0.70**	**6.13**		**11.71**	**0.80**	**7.01**
**RMSE**			**0.89**				**1.00**	
*P* value of paired *T* test*					0.65			

**Table 2:** Area-based statistics of the spatial distribution maps of PM_2.5_ concentration

	Max value (μg/m^3^)	Min value (μg/m^3^)	Ave. value (μg/m^3^)	Information Entropy
OK interpolated	12.93	10.08	11.21	3.63
LUR model Simulated	13.52	9.57	9.86	7.79
Observed	14.24	9.87	11.65	-
